# The impact of selected abiotic factors on *Artemia* hatching process through real-time observation of oxygen changes in a microfluidic platform

**DOI:** 10.1038/s41598-023-32873-1

**Published:** 2023-04-19

**Authors:** Preyojon Dey, Terence M. Bradley, Alicia Boymelgreen

**Affiliations:** 1 Department of Mechanical and Materials Engineering, Florida International University, 10555 W Flagler St, Miami, FL 33174 USA; 2 Department of Fisheries, Animal and Veterinary Science, University of Rhode Island, Kingston, RI 02881 USA

**Keywords:** Ecology, Environmental sciences, Engineering, Ecology

## Abstract

Current studies on abiotic impacts on *Artemia*, a crustacean which is widely used in aquaculture, and ecotoxicology, often focus on endpoint analysis (e.g., hatching rates, survival). Here, we demonstrate that a mechanistic understanding can be obtained through measurement of oxygen consumption in real-time over an extended time period in a microfluidic platform. The platform enables high level control of the microenvironment and direct observation of morphological changes. As a demonstration, temperature and salinity are chosen to represent critical abiotic parameters that are also threatened by climate change. The hatching process of *Artemia* consists of four different stages: hydration, differentiation, emergence, and hatching. Different temperatures (20, 35, and 30 °C) and salinities (0, 25, 50, and 75 ppt) are shown to significantly alter the duration of hatching stages, metabolic rates, and hatchability. Specifically, the metabolic resumption of dormant *Artemia* cysts was significantly enhanced at higher temperatures and moderate salinity, however, the time needed for this resumption was only dependent on higher temperatures. Hatchability was inversely related to the duration of the differentiation stage of hatching, which persisted longer at lower temperatures and salinities. The current approach of investigation of metabolism and corresponding physical changes can be employed to study hatching processes of other aquatic species, even those with low metabolic rate.

## Introduction

The rising concentration of greenhouse gases caused by anthropogenic activity is modifying the global climate, and one of the immediate results is higher ambient temperature^1–3^. The oceans around the world, acting as heat sinks, absorb the bulk of this heat^4,5^. Thermal expansion of the oceans as a result of increased heat content, as well as glacier melting, produces a rise in ocean volume, which has a direct effect on ocean water salinity^6,7^ that is further exacerbated by changes in the global water cycle^8,9^. The effects of variations in these parameters on reproduction and health may influence the abundance and distribution of different aquatic species. The precise mechanisms of action of different changing environmental factors, such as abiotic factors^10,11^, and environmental toxicants^12,13^ on the phenotypic response of diverse species have been investigated. Here, we aim to obtain a mechanistic understanding of the impacts of changes in the abiotic environment on the hatching process of *Artemia,* commonly referred to as brine shrimp, through real-time monitoring. *Artemia* is a popular live feed used in aquaculture due to its high nutritional density, ease of culture and relatively small size which makes this species optimal for facilitating feeding of marine larvae with small mouth gape^14,15^. It is also extensively utilized as a model organism in biochemical, physiological, genetic, ecological and ecotoxicology studies^16–18^. Despite the fact that *Artemia* live in a hypersaline environment which is their only defense mechanism against predators^19,20^, the mechanistic understanding of the effects of abiotic factors on the hatching process of *Artemia* can potentially provide insights into the effects of altering temperature and salinity on the hatching of other widely spread crustaceans in the marine environment, such as Copepods.

*Artemia* females develop a diapause cyst with no detectable metabolic activity during oviparity mode of reproduction (endogenous diapause)^21^. In this dormant stage, cysts may be dehydrated through air drying or osmotic water removal, at which point the cysts are quiescent and can survive up to 28 years^22^. The metabolism of the cysts can be resumed -and hatching initiated—when the environmental conditions are favorable^23^, with the primary abiotic environmental parameters affecting hatchability being water temperature and salinity^17,24,25^. A number of previous studies have investigated the impact of these abiotic environmental parameters on *Artemia* hatching^26–31^, however, the evaluation was limited to the measurement of the hatching rate at the endpoint of the experiment. For instance, Kumar et al. observed that optimal hatching performance of *Artemia* occurred at 29 °C and 29 parts per thousand (ppt) salinity^26^ while Sharahi et al. found the optimal conditions to be 27–28 °C and 35 ppt, respectively^30^. Hasan et al. showed that the maximum proportion of *Artemia* hatched when the salinity and temperature were 30 ppt and 24 °C, respectively, and that the hatching rate decreased as the temperature rose from 24 to 32 °C while the salinity ranged between 20 and 40 ppt^27^. Ahmed et al. reported that the optimal salinity for hatchability is 20 ppt^31^. In another study done by Bahr et al. on the influence of salinity on hatching, the optimal hatchability was found at salinities of 60 ppt and temperatures around 30 °C^28^. The variation in optimal parameters could be due to differences in test settings (e.g., light exposure^25,30,32^) or variance in environmental conditions (container size, temperature/salinity gradients in bulk media).

This work aims to unify environmental conditions and extend the understanding of the effects of abiotic parameters beyond the endpoint metric of hatching rate to an in-depth, mechanistic analysis of their impacts on the hatching process of *Artemia*. The four distinct stages of hatching of *Artemia*—(1) hydration, (2) differentiation, (3) emergence, and (4) hatching^33^ can be differentiated based on the morphological changes and metabolism^34,35^. In this study, we have integrated an optical oxygen sensor in a microfluidic “aquarium” to monitor rate of oxygen consumption, also referred to as routine metabolic rate^36^, in real-time throughout the entire hatching process, while an optical microscope is employed to capture photomicrographs of the hatching cysts at regular time intervals. Previously metabolism of *Artemia* cysts^37^ and those of other small crustaceans such as *Acartia tonsa*^38,39^, and *Leptodora kindti*^40^ have been investigated by using different respirometry techniques. The relatively small size of the microfluidic platform used in this study can decrease the signal-to-noise ratio^41–43^ of the oxygen sensor and enable the correlation of sensor data with microscope imaging data. The platform can also more readily maintain uniform environmental conditions^44–46^ using off-chip programmable controllers as compared to bulk systems (such as beakers, fish tanks) used in contemporary studies. Recently, microfluidic and millifluidic lab-on-a-chip platforms have been used for pharmaceutical applications including drug testing on cells^47^, biological studies on species such as *C. elegans*^48–52^, and zebrafish^53–55^. Moreover, in the present work, the accuracy of the end point analysis of the hatching rate is also enhanced through the automated transfer -minimizing contamination and human error^56–59^—of the entire tested sample to a counting chip with sieve-like structures that can be imaged in its entirety and processed with image analysis rather than relying on a batch sample and manual counting methods.

## Experimental

### *Artemia* cyst

**Figure 1:**
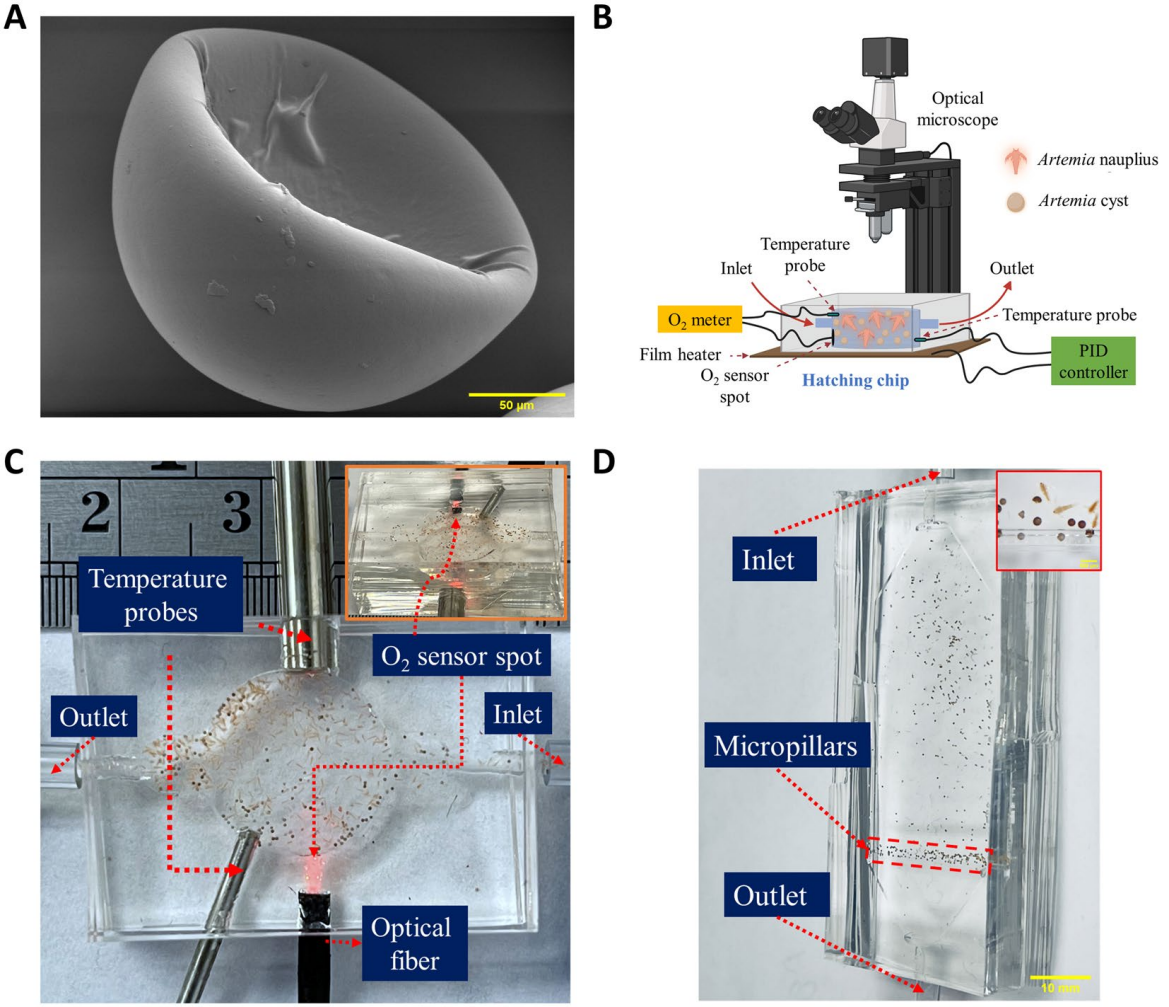
Experimental setup. (**A**) Scanning electron microscopy (SEM) shows the cup-like structure of the as-received *Artemia* cysts at 450X magnification. (**B**) Schematic overview of the microfluidic platform to study the effect of abiotic environmental parameters on the hatching performance of *Artemia*. (**C**) Hatching chip with integrated O_2_ sensor and temperature probes connected (scale bar = 5 mm) (Position of O_2_ sensor spot is shown in inset) (**D**) Counting chip with sieving-like structures made of PDMS micropillars at the outlet (scale bar = 10 mm) [Cysts and nauplii entrapped at the sieving structures are shown in inset (scale bar = 500 µm)].

*Artemia* cysts were purchased from Brine Shrimp Direct and stored at 4 °C as recommended by the vendor until one day prior to the hatching studies, at which point they were transferred to room temperature to allow for gradual temperature adaptation. Figure 1A shows the scanning electron microscopy (SEM) (JEOL FS-100) images of the as-received *Artemia* cysts. The cysts are cup-shaped in structure, and the diameter of the circular structure of the cup ranges between 180 and 260 µm.

### Microfluidic platform for hatching

The hatching studies of the *Artemia* cysts were performed on a microfluidic platform (Fig. 1B). The platform consisted of a hatching chip, which has a cylindrical chamber (diameter of 13 mm, height of 3.55 mm) connected to an inlet and outlet (height of 1.7 mm, width of 4 mm) (Supplementary Fig. 1A). The total volume of the hatching chip is ~ 563 µL. Rounded corners (fillets) were incorporated at the inlet and outlet to ensure cysts were not trapped in the chamber corners during insertion at the start of the hatching and the transfer of the cysts and nauplii at the end of the experiment. Dead volume was minimized through simulating the fluid flow in ANSYS FLUENT (Supplementary Fig. 1B). Figure 1C shows the hatching chamber used for this study. The chip was composed of polydimethylsiloxane (PDMS) and fabricated by casting PDMS on a mold (Supplementary Fig. 1C) prepared using a 3D printer (MAX X-43, Asiga) and cured overnight at 60 °C in a gravity convection oven (model: 10GC, Quincy Lab). The hatching chip has two layers. The top layer has the chamber and channel structures, and the bottom layer is a blank PDMS slab. Both top and bottom layers of the hatching chip were bonded using corona treatment (BD-20AC, ETP), and subsequent heating at 60 °C for overnight. Before bonding the layers, several holes were made in the top layer using biopsy punches for the inlet, outlet, and sensors. An O_2_ sensor spot (OXSP5, Pyroscience) was bonded to the PDMS top layer inside the chamber using silicone glue (Spglue, Pyroscience). An optical fiber was connected exactly behind the O_2_ sensor spot through a hole in the PDMS chip. The optical fiber was connected to an external optical oxygen meter (FireSting^®^-O_2_, Pyroscience). The oxygen meter was composed of a light-emitting diode (LED) and a photodiode that both stimulate and detect the oxygen-sensitive spot oxygen-dependent luminescence emission and measures the dissolved oxygen concentration of the water in the chamber every second during hatching. A temperature probe (Pt100, Pyroscience) was also connected to the chip to detect the temperature of the water inside the chip and thus compensate the dissolved oxygen concentration readings for any fluctuation in temperature. To minimize variation, the chip was placed on top of a film heater (PI film heater-24 V, Icstation) connected to a proportional–integral–derivative (PID) controller (6–30 V DC Electronic Thermostat Controller, DROK) which controlled the temperature of the water inside the hatching chip according to the set temperature (20, 25 and 30 °C). A variable voltage DC power adapter (ALP002, KEJIESHENG) was used to power the PID controller, and a thermocouple probe (TC 10K, QINGDAO) was connected to it for measuring the water temperature inside the chip. The PID controller adjusts the input power according to the probe measurement to maintain a maximum variation of 0.1 °C from the set temperature. The hatching chip with the embedded heater was placed under a digital stereo optical microscope (SE400-Z, Amscope) equipped with a digital camera (MD500, Amscope) to capture a photomicrograph of the chip every five minutes during hatching. During the entirety of the hatching process, a light-emitting diode (LED) light (1W, Amscope) was kept on continuously due to its effect on hatching^26,32^.

### Hatching of *Artemia*

Prior to the start of the experiment, the water for hatching was prepared by adding varying amounts of commercially available sea salt (Fluval Sea, pH: 8.1–8.2) to deionized water (DIW) (prepared and filtered (filter pore size = 200 nm) using Barnstead Smart2Pure Water Purification System, Thermo Scientific) to produce artificial saltwater with 0, 25, 50, and 75 parts per thousand (ppt) salinities which were mixed in a test tube using a vortex mixer (Vortex genie 2, Scientific Industries). Thorough mixing of the saltwater solution allows uniform distribution of the dissolved oxygen and salt. The weight of *Artemia* cysts was measured using a digital precision balance (Bonvoisin) and mixed carefully with the saltwater solution so that the cyst concentration was 5 g/L. The cyst solution was immediately inserted inside the hatching chip until the chamber was filled. The inlet and outlet of the chip was then closed using flexible tubes and binder clips to prevent air infiltration inside the chip. Hatching experiments were conducted for 24 h from the time cysts were immersed in the saline solutions. The hatching experiments were performed in triplicate for each temperature (20, 25, and 30 °C) and salinity (0, 25, 50, and 75 ppt) condition.

### Rate of oxygen consumption and duration of different stages

The different hatching stages of *Artemia* and the transition between them can be identified through changes in depletion in dissolved oxygen concentration (DDOC) of the water in the closed hatching chip as a result of the oxygen consumption by the cysts, as measured by the on-chip oxygen sensor and the photomicrographs taken by the optical microscope. This provides information regarding the oxygen consumption and duration of the various stages of hatching. The results of the oxygen consumption are normalized by taking into account the rate of oxygen concentration (ROC) at each stage because the absolute value and the duration of the various stages of hatching vary with temperature and salinity. ROC can be also considered as standard metabolic rate as mentioned in the literature^36^. ROC was calculated using the following formula

1 $$Rate\;of\;oxygen\;consumption\;(ROC) = \frac{{DDOC\;at\;any\;stage}}{{The\;duration\;of\;the\;respective\;stage}}$$

At each hatching stage, the duration and the corresponding ROC were measured—with the exception of certain temperatures and salinities, at which the emergence and hatching stages were not complete in the hatching period of 24 h (See “Effect of water salinity and temperature on the duration of different stages of hatching”).

### Counting chip and automatic hatching performance calculation

The depth of the hatching chip (3.55 mm) was designed to ensure the suspending fluid had sufficient dissolved oxygen for hatching and space for the nauplii to swim. This depth allowed multiple nauplii and cysts in the same vertical plane, which precluded accurate counting of nauplii and cysts. Hence, a counting chip (Fig. 1D) was designed with a shallow depth (800 µm) chamber such that nauplii and cysts were restricted to a single monolayer. The chip had an inlet and an outlet. The outlet contained PDMS micropillars which acted as sieving-like structures (Fig. 1D inset) and allowed only the water to flow through the channel, preventing the nauplii and cysts in the chamber from escaping. The chip had two layers: the top layer had a chamber, micropillar structures, inlet, and outlet and was constructed of PDMS cast on a 3D printed mold (Supplementary Fig. 2A). The bottom layer was a blank PDMS slab. Both layers were bonded using corona treatment and subsequent heating, as described above. At the inlet and outlet of the counting chip, the height is higher than that of the chamber (1.7 mm vs. 800 µm) and designed with a fillet radius of 900 µm to allow smooth flow of *Artemia* cyst and nauplii through the inlet and outlet. Supplementary Fig. 2B displays the results of an ANSYS Fluent simulation of the fluid flow through the chip, demonstrating the absence of any dead volume. After 24 h, the hatched nauplii and hatched/unhatched cysts in the hatching chip were transferred to the counting chip automatically in a 30% methanol solution at a 100 µL/min flow rate by syringe pump (Pico plus 11 elite, Harvard Apparatus). The outlet of the hatching chip and the inlet of the counting chip were connected by a poly(tetrafluoroethylene) (PTFE) tube, and the inlet of the hatching chip is connected to the syringe mounted in syringe pump via a flexible Tygon tube. *Artemia* nauplii swim rapidly after hatching and the 30% methanol solution in DIW was used to euthanize the nauplii to obtain clear images in the counting chip. A digital camera (D3100, Nikon) was used to capture images of the nauplii, and cysts present in the hatching chip. Images were processed in ImageJ where the number of cysts and nauplii were counted based on their circularity. If any object had a circularity greater than or equal to 0.9, it was considered to be a cyst (hatched or unhatched), and any object in the image with lower circularity (less than 0.9), was considered to be nauplii (Supplementary Fig. 3). The hatching rate was calculated using the following formula

2 $$Hatching\;rate= \frac{Total\;number\;of\;nauplii}{Total\;number\;of\;cysts}$$

### Statistical analysis

OriginPro (ver. 2022b, OriginLab) was used for all statistical analysis. Data are represented as the mean ± standard deviation. As mentioned previously in “Rate of oxygen consumption and duration of different stages”, at certain temperatures and salinities, the emergence and hatching stages did not complete within 24 h. Thus, for these stages, the significance of the data was determined using a one-way analysis of variance (ANOVA) followed by a Bonferroni post-hoc test. However, for the first two stages (hydration and differentiation) and endpoint hatching rate, data were obtained at all temperatures and salinities, and the significance of the data within and between groups was determined using a two-way ANOVA with Bonferroni post-hoc test. The ANOVA tables are included in Supplementary Table 1. Prior to performing the ANOVA tests, the data were examined for normality using a normal quantile–quantile plot with a confidence level of 95%. Analysis of the correlation between different results was performed using the Pearson correlation coefficient. Data were considered significant in all statistical analyses if *p* < 0.05.

## Results and discussion

### On-chip detection of oxygen consumption and morphological changes in cysts during the hatching process

**Figure 2:**
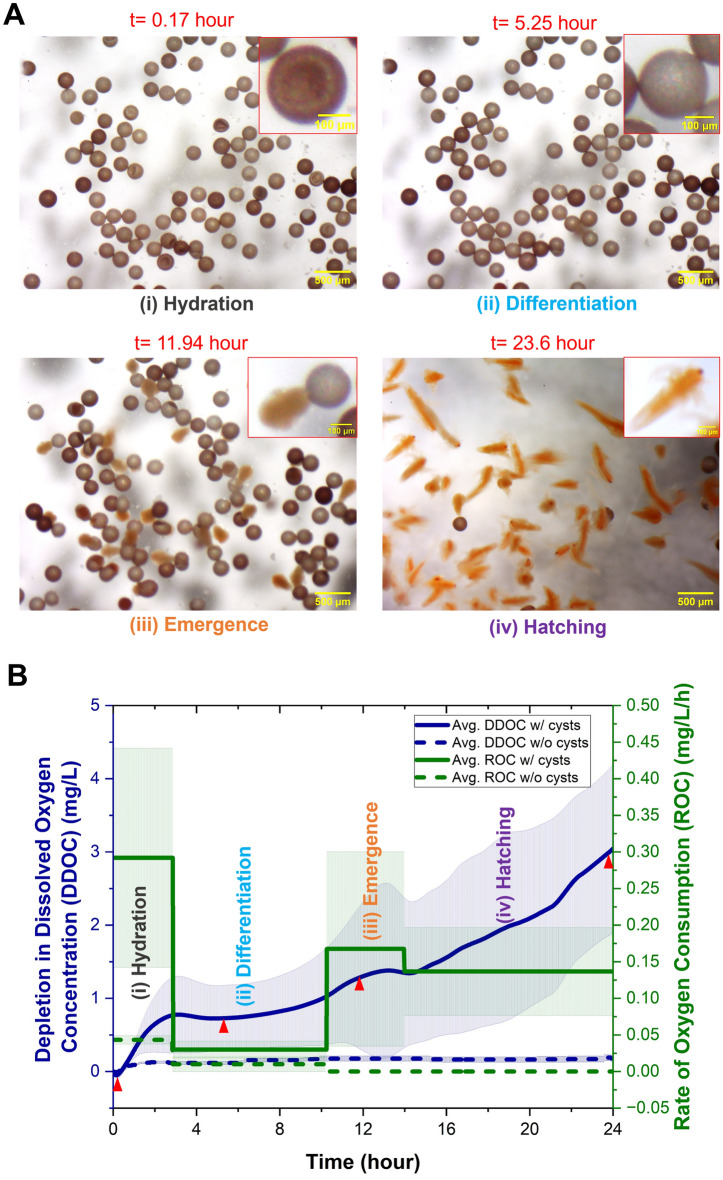
Photomicrographs and O_2_ sensor readings at different stages of hatching. (**A**) Photomicrograph at different stages of hatching obtained from the optical stereomicroscope (scale bar = 500 µm). The inset shows the morphology of the cysts/nauplii at different stages (scale bar = 100 µm). (**B**) On-chip detection of the depletion in dissolved oxygen concentration (DDOC) when there were *Artemia* cysts (blue solid line) and no cysts (control-blue dashed line) in the hatching chip at 25 °C and 25 ppt. Rate of oxygen consumption (ROC) at different stages was represented by green lines (solid line- with cysts, dotted line-control). The bold lines in the graph show the average and the shaded area shows the standard deviation. Red triangles in (**B**) represent the DDOC at the time points corresponding to the photomicrographs presented in (**A**).

As *Artemia* cysts hatch, their oxygen consumption and morphology undergo changes. As described in the methods section, these changes were recorded using an on-chip oxygen sensor and an optical microscope. Figure 2A depicts photomicrographs of various stages of hatching. Figure 2B depicts the depletion in dissolved oxygen concentration (DDOC) of the water inside the hatching chip. When there were cysts within the hatching chip, as the cysts consumed oxygen throughout the hatching process, the concentration of dissolved oxygen in the water decreased and thus DDOC increased (blue solid line). The rate of oxygen consumption (ROC) at each stage, as estimated by Eq. (1), is also depicted in Fig. 2B (green solid line). When the hatching chip contained only artificial saltwater but no cysts (control experiment), the DDOC (blue dotted line) and ROC (green dotted line) were almost negligible. It is important to note that although the hatching chip material PDMS is permeable to oxygen^60,61^, comparison of the DDOC with cysts within the chip to the DDOC without cysts (control) indicates that the rate of oxygen consumption at all stages is significantly larger than permeation thereby supporting the use of the present setup to qualitatively compare the DDOC under different environmental conditions. We note that since the *Artemia* cysts used for hatching in this study were not sterilized, there is a possibility that they may contain microorganisms that consume some of the dissolved oxygen. The effects of cysts and the potential presence of microorganisms on the DDOC were not distinguished in this study, and further investigation is required. However, as the control experiment indicates (dotted lines in Fig. 2B) negligible changes in DDOC over the 24-h time period, it can be concluded that neither the hatching chip nor potential microorganisms in the artificial saltwater itself contributed to changes in the DDOC.

Immersion of the cysts in water starts the hydration stage, in which the cysts inflate and develop into a spherical (rather than cup like) shape [Fig. 2A(i)]. As the cysts imbibe water, energy metabolism, RNA, and protein synthesis begin within a short period^34,62^. As energy metabolism is reactivated, respiratory activity increases dramatically, as evidenced by the increased ROC (Fig. 2B). At the end of the hydration stage, the majority of the cysts are spherical in shape and differentiation commences. Also, at this stage, there is no cell division and no increase in DNA^34,62^ resulting in an almost constant and low oxygen requirement and thus lower ROC (Fig. 2B). In the third stage (emergence), the cyst shell starts to fracture due to the increased turgor pressure within resulting from an increased amount of glycerol, and the embryo starts to emerge from the broken shell within a hatching membrane [Fig. 2A(iii)]^35^. The emergence stage is highly active and needs additional energy, resulting in an increase in DDOC and ROC (Fig. 2B). Finally, at the last stage (hatching), the *Artemia* embryos leave the cyst shell and hatching membrane and begin to swim [Fig. 2A(iv)]. At this stage, oxygen demand lowered again, as detected by a comparative decrease in ROC (Fig. 2B).

### Effect of water salinity and temperature on the duration of different stages of hatching

**Figure 3:**
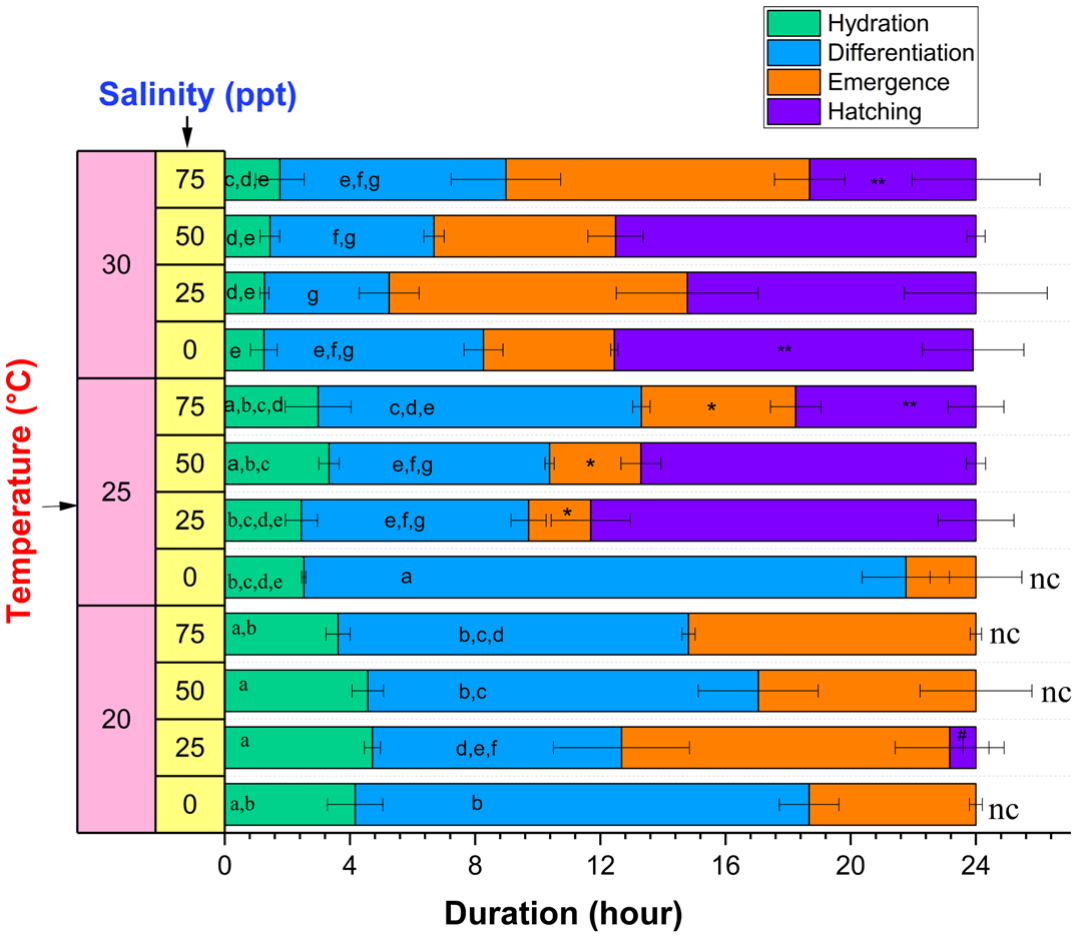
Duration of different stages of hatching at different water salinities and temperature. Values are expressed as mean ± standard deviation (n = 3). In each category (hydration, and differentiation stage duration), means that do not share the same letter are significantly different from each other (two-way ANOVA with Bonferroni post-hoc test, *p* < 0.05). The hatching stages which were not complete within 24 h were not statistically analyzed and marked with “nc”. * and ^#^ denote significantly different emergence and hatching stage duration, respectively, at a temperature as compared to the other tested temperatures (one-way ANOVA with Bonferroni post-hoc test, *p* < 0.05). ** denotes significantly different hatching stage duration between two different tested salinities (one-way ANOVA with Bonferroni post-hoc test, *p* < 0.05).

The duration of each stage of hatching at various temperatures and salinities is presented in Fig. 3. Overall, we note that at the lowest temperature (20 °C) and/or lowest salinity (0 ppt) in our tested range, the 24-h time period was insufficient to complete the hatching due to the prolonged duration of each of the first three stages, with the exception of 20 °C at 25 ppt and 0 ppt at 30 °C. In line with this, the duration of the hatching stage (defined as the time remaining at the end of the emergence stage) was found to decrease considerably when salinity increased to 75 ppt at 25 and 30 °C. At 20 °C (where hatching stage was observed at only 25 ppt salinity), the hatching stage duration was reduced significantly compared to that at all other temperatures studied, regardless of salinity.

Recognizing that during hydration (green bars, Fig. 3), the dry *Artemia* cysts absorb water by osmosis^63^, the effect of temperature and salinity can be modelled by Van’t Hoff’s law^64^

3 $$\Delta \pi =RT\Delta C$$

where Δπ is the difference in osmotic pressure, R is the universal gas constant, T is the temperature, and ΔC is the difference in solute concentrations. As the temperature (T) increases, so does the rate of osmosis, corresponding to a decrease in the hydration duration as illustrated in Fig. 3. Supplementary Fig. 4A shows the significant negative correlation between the hydration duration and temperature determined by the Pearson correlation coefficient (r = − 0.9) and a linear fit between these parameters (R^2^ = 0.99) (Supplementary Fig. 4B). At the same time, the increase in water salinity at any temperature can decrease the difference in internal and external solute concentrations (ΔC) of cysts, which should decrease osmotic pressure with an increase in the hydration duration. However, the experimental results indicate that changes in the salinity did not significantly affect the hydration duration. In contrast, the duration of differentiation (blue bars, Fig. 3) decreased significantly when temperature was increased or salinity was increased from 0 to 25 ppt (does not vary significantly beyond 25 ppt) at any temperature, except 30 °C. To understand this, we note that through the differentiation stage, the cyst reaches the emergence stage, when the increasing turgor pressure induced by the synthesis of glycerol from trehalose causes the shell to begin to fracture. A rise in temperature enhances glycerol synthesis^63^, which in turn shortens the period of differentiation. Increasing salinity also increases the glycerol level^35^, and may decrease the differentiation period. However, as water salinity rises, the external environment becomes hypertonic, and there is a chance of cellular water loss due to the exosmosis process, which could lengthen the period of differentiation at higher salinity. The competition between these two opposing factors could explain why there was no significant difference in differentiation stage duration beyond 25 ppt salinity. Interestingly, the emergence stage duration (orange bars, Fig. 3) suggests an optimum temperature of 25 °C with a significant decrease in duration, compared to that at 20 and 30 °C. Although temperature helps to increase the osmotic pressure and thus reduces the hydration and differentiation stage length, once the emergence stage starts, and the embryo emerges within the hatching membrane, it appears to have less tolerance for higher temperatures. Hence, an intermediate temperature of 25 °C was more favorable for the emergence stage and required less time for completion.

### Effect of water salinity and temperature on oxygen consumption during hatching

Further understanding of the hatching process is obtained by directly examining the depletion in dissolved oxygen concentration (DDOC) of water in the hatching chamber for varying temperatures and salinities. Supplementary Fig. 5 shows the average DDOC curves of triplicate experiments performed at each temperature and salinity condition. Overall, the results indicate a positive correlation between hatching duration and DDOC for salinities greater than zero.

**Figure 4:**
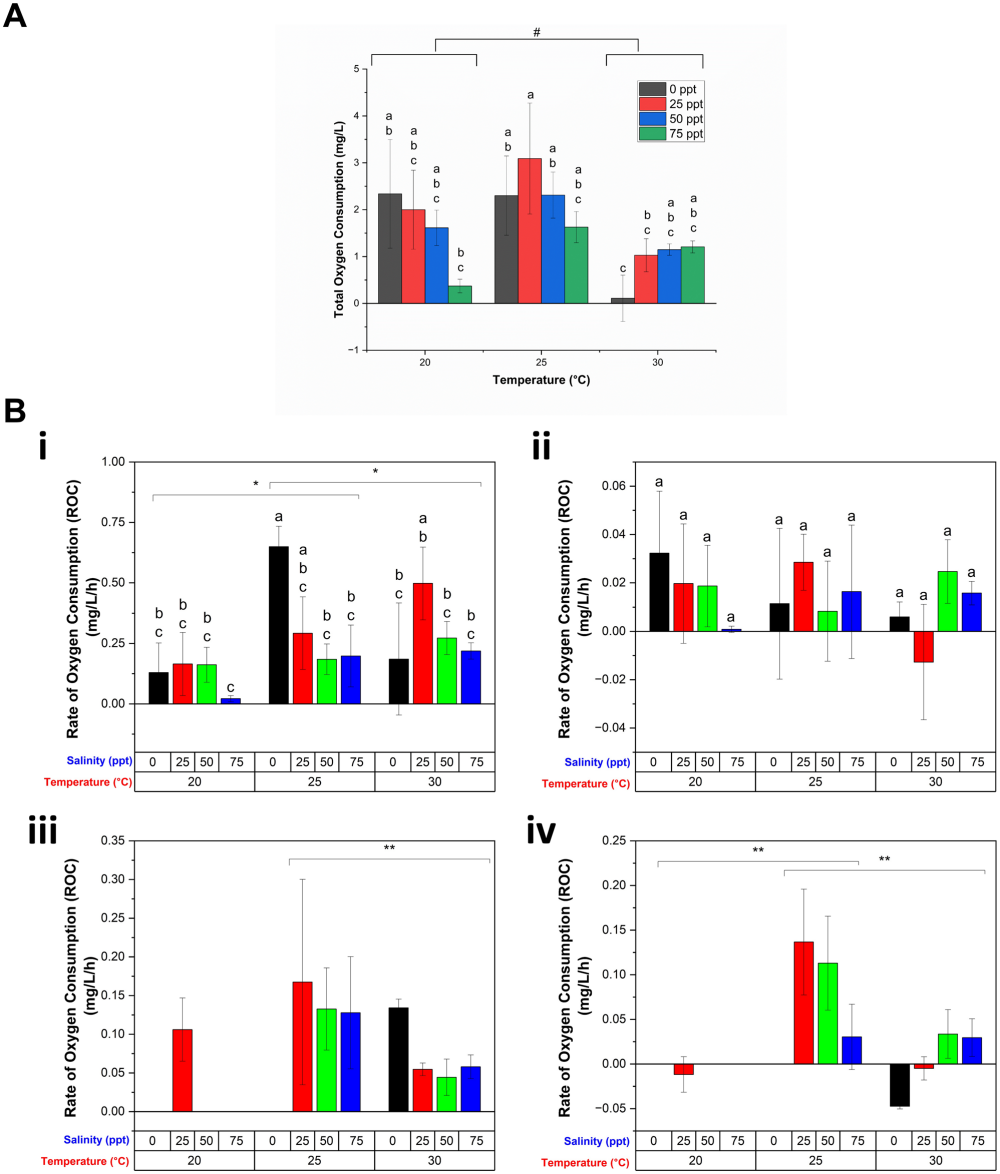
Oxygen consumption of the *Artemia* cysts under different temperatures and salinities. (**A**) Total oxygen consumption, or total DDOC throughout the entire hatching process under different temperature and salinities. (**B**) Rate of oxygen consumption (ROC) at different stages of hatching: (i) hydration, (ii) differentiation, (iii) emergence, and (iv) hatching stage. Values are expressed as mean ± standard deviation (n = 3). In each category of data (total oxygen consumption, or ROC in hydration and differentiation stages), means that do not share letters above the columns are substantially different from each other (two-way ANOVA with Bonferroni post-hoc test, *p* < 0.05). At different tested temperature, statistically significant ROC was indicated by * (two-way ANOVA with Bonferroni post-hoc test, *p* < 0.05) and ** (one-way ANOVA with Bonferroni post-hoc test, *p* < 0.05) respectively, whereas # indicates statistically significant total oxygen consumption (two-way ANOVA with Bonferroni post-hoc test, *p* < 0.05).

The total DDOC, or total oxygen consumption, for the 24-h hatching period is shown in Fig. 4A, indicating the presence of an optimum temperature (25 °C), because total oxygen consumption is higher at this temperature regardless of salinity. The total oxygen consumption decreased significantly as the temperature increased from 20 to 30 °C irrespective of any salinity. Salinity lowered the total oxygen consumption significantly only when it was increased from 25 to 75 ppt, regardless of temperature; however, there was no significant change when the salinity was altered at 30 °C, suggesting a significant interaction between temperature and salinity. Based on the weight of the dry initial cysts used, this total oxygen consumption ranged from 5.81 × 10^–7^ to 3.47 × 10^–5^ mg O_2_/h/mg dry wt, with varying temperature and salinity, which is comparable to the oxygen consumption of *Artemia*^37^, and other crustacean eggs, such as *Anomalocera patersoni*^65^ and *Pontella mediterranea*^66^ reported in earlier studies.

A more detailed understanding of the effects of temperature and salinity on the hatching process is obtained through consideration of oxygen consumption at each of the four stages of hatching. Regardless of salinity, ROC increased significantly during hydration when temperature was increased from 20 °C [Fig. 4B(i)]. ROC also significantly decreased with increased salinity between 25–75 ppt which reflects lower activity in accordance with the longer duration (Fig. 3). On the other hand, although both salinity and temperature decreased duration of differentiation, the metabolic rate was not significantly affected [Fig. 4B(ii)]. During the emergence stage, ROC was found to be significantly affected by temperature, but not salinity at *p* = 0.05 level (one-way ANOVA), with ROC being significantly decreased when temperature was increased from 25 to 30 °C (Bonferroni, *p* < 0.05, Fig. 4B(iii)). We note that the statistical significance of the data was assessed using one-way ANOVA with Bonferroni post-hoc test due to incomplete emergence in some experimental conditions (Fig. 3). The decreased oxygen consumption with increased temperature may indicate physiological stress. Finally, in the hatching stage, ROC decreased as compared to that at the emergence stage, and it was maximum at 25 °C irrespective of salinity, and salinity has no effect on ROC at this stage. A similar increase in energy demand during emergence and a decrease in that following hatching was observed in other crustacean species, such as *Acartia tonsa*^67^, *Cherax quadricarinatus*^68^, and *Cancer pagurus*^69^. In our study, the oxygen consumption per unit of dry weight of the *Artemia* cysts in emergence stage ranged from 7.98 × 10^–7^ to 2.5 × 10^–5^ mg O_2_/h/mg dry wt with varying temperatures and salinities. Based on the upper limit, the oxygen uptake during the emergence of *Artemia* cyst is approximately 5.54 times lower than big crustacean *Cancer pagurus*^69^ and 0.43 times higher than small crustacean *Pontella mediterranea*^66^. Moreover, a negative ROC was observed at the hatching stage under certain temperature and salinity conditions. At 25 ppt salinity at 20 °C and 30 °C, this could be due to very low oxygen consumption of hatched *Artemia* and possible permeation of a very low amount of oxygen through PDMS (see Fig. 2B-control experiment). However, at 0 ppt/30 °C, it was observed that all the nauplii died following hatching (no mobility) potentially due to the combined effect of zero salinity and high temperature, and for unknown reasons, the oxygen concentration of the water increased during this time. One possible explanation could be that during the hatching process, some cysts were observed to release air bubbles that may have initially been trapped within the cyst shell. Live nauplii would consume this extra oxygen from air bubbles; but, in this instance, because there were no living nauplii present, the oxygen content of the hatching water increased slightly. However, the exact reason for this increased oxygen content is unknown, and requires further investigation.

### Effect of water salinity and temperature on hatching rate

**Figure 5:**
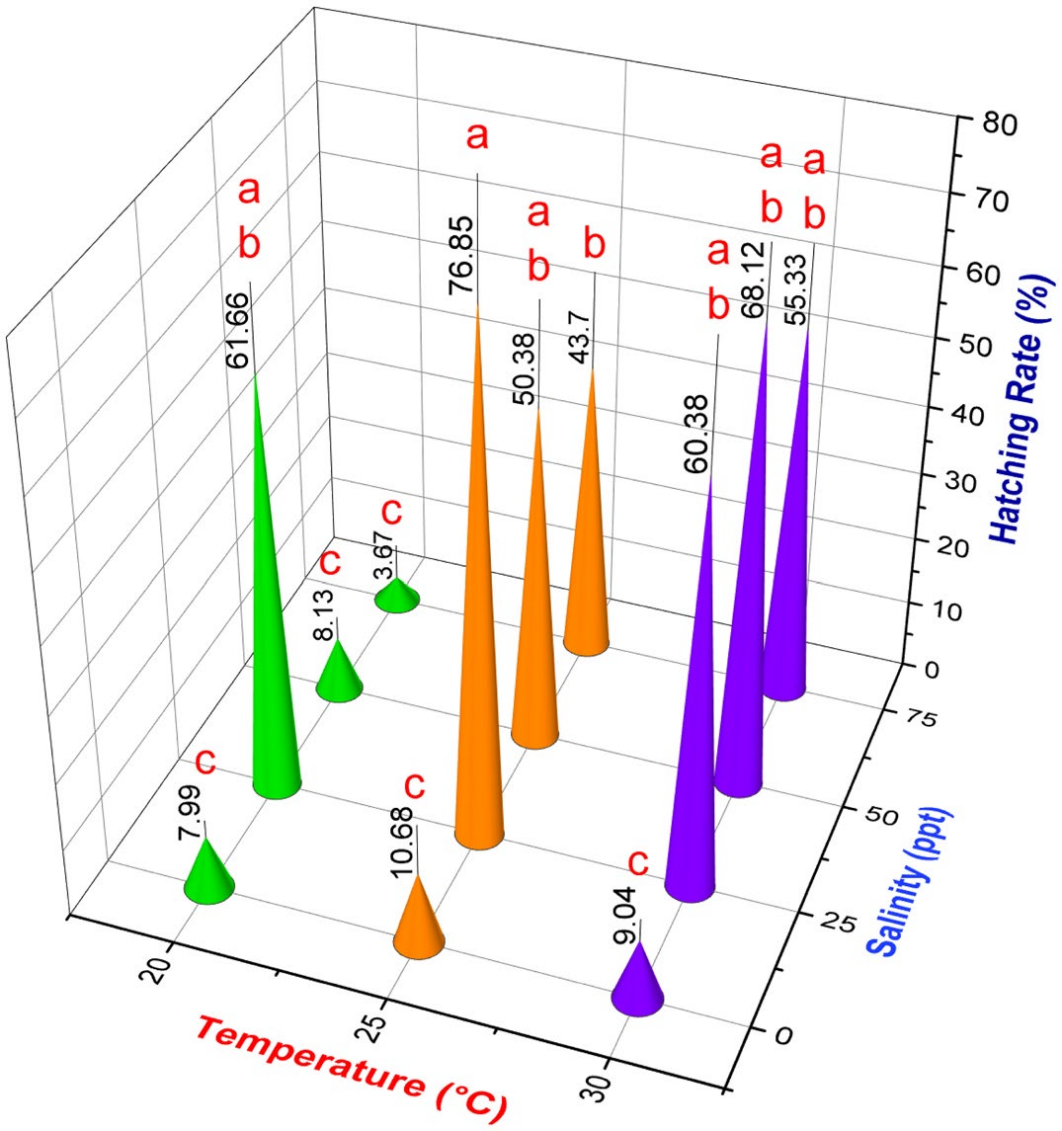
On-chip hatching rate calculation of *Artemia* at different water salinities and temperatures. Values are expressed as mean ± standard deviation (n = 3). Means that do not share the same letter above the columns are substantially different from each other (Two-way ANOVA with Bonferroni, *p* < 0.05).

Figure 5 illustrates the hatching rate (estimated using Eq. (2)) of *Artemia* cysts in our platform at varying temperatures and salinities. In accordance with previous work, the results indicate the existence of optimal conditions^27,30^. Specifically, the hatching rate increased dramatically when the temperature rose from 20 to 25 °C, but there was no significant change as the temperature rose further. Similarly, the hatching rate increased when salinity was increased from 0 to 25 ppt at 20 and 25 °C, and 0–50 ppt at 30 °C, but subsequently reduced with increased salinity. A maximum hatching rate was observed at 25 °C temperature and 25 ppt water salinity (76.85 ± 14.22%) in accordance with the measured maximum ROC [Fig. 4(iv)]. Moreover, the measured ROC indicated that the very low hatching rates at zero salinity and 20 °C do not necessarily reflect hostile conditions (since we do observe activity) but that the 24-h duration is insufficient.

### Correlation of respiration behavior, duration of different hatching stages and hatching rate

The present approach, in which the entire hatching process is monitored in real time, uniquely advances the understanding of the relationships between the abiotic parameters, stage characteristics (duration, ROC) and hatching rate. To quantify these parameters, we implemented a Pearson correlation. We first note that although ROC was significantly affected by temperature, salinity, or both, only ROC at the hatching stage had a statistically significant association (positive correlation) with the hatching rate. On the other hand, only differentiation duration is significantly correlated with hatching rate (negative correlation).

Negative correlations between hatching rate and hydration, and differentiation stage duration and a positive correlation between hatching rate and hatching stage duration were observed. The hatching rate was maximum when the temperature and salinity were 25 °C and 25 ppt, respectively. At lower or higher temperature or salinity among the tested conditions, the hydration and differentiation stage took longer, and hatching stage was shorter. Similarly, oxygen consumption correlates positively with hatching rate at the hydration and hatching stage, but negatively with hatching rate at the differentiation stage. At 25 °C temperature and 25 ppt salinity, the oxygen consumption was comparatively high at the hydration stage, lower during the differentiation stage, and very high at the hatching stage. This pattern was reversed at very low or very high tested temperatures or salinities. These findings show that temperature and salinity significantly affect the whole hatching process. Highest hatchability is obtained at that favorable temperature and salinity condition, when the majority of the cysts become hydrated and regain a higher metabolic rate in a shorter period of time during the hydration stage, expend less energy during the differentiation stage and complete it more quickly. At this optimal condition, cysts will spend a longer time in the hatching stage, resulting in a larger number of cysts hatching and greater oxygen consumption.

## Conclusion

In this work, we have demonstrated that measurement of metabolic rates in real-time and the corresponding morphological changes may offer in-depth, mechanistic understanding of *how* abiotic parameters affect the hatching process of *Artemia*. Focusing on temperature and salinity (two significant environmental parameters that are also susceptible to climate change), we measured the respiration behavior, progression of hatching as well as the resulting number of hatched *Artemia* under distinct temperature and salinity levels throughout a predetermined time period. Although *Artemia* habitats are limited by hypersalinity, this study can shed light on the likely impacts of temperature and salinity on the hatching process of other more widely spread crustacean species.

In accordance with previous studies, extreme salinities (low or high) and low temperatures are observed to inhibit *Artemia* hatching verifying the existence of optimal hatching conditions^26–31^. However, while these previous studies primarily focus on endpoint measurements of the hatching rate, here we have established relationships between the hatching rate and the progression of hatching and respiration behavior throughout the process. For example, we demonstrate that a key factor affecting the hatchability of *Artemia* in a 24 h time period is the duration of the differentiation stage, which is found to be inversely proportional to the hatching rate. Overall, the duration of differentiation (blue bars, Fig. 3) decreases with both increased salinity and temperature, except at very high salinities. Based on an understanding of the differentiation stage, it is possible that this relationship is connected to increased glycerol production that accelerates shell fracture characterizing the onset of emergence. Although validation of this hypothesis is left to future work, we illustrate that the current experiments highlight key areas of focus towards understanding the complex relationship between environment and aquatic animal health. A crucial link between temperature, salinity and the reactivation of dormant cyst metabolism was also clearly demonstrated by comparing hydration progression with Van't Hoff's law.

The present experimental approach was enabled through development of a novel microfluidic platform with integrated oxygen sensor, in which physical changes in the *Artemia* cysts could also be recorded using an optical microscope. Precise environmental control is obtained using a heater with a feedback loop and an automated counting chip is designed to eliminate errors associated with the lack of stringent environmental control and manual calculation of hatchability. As well as offering a more in-depth look at biological processes, widespread use of automated systems with precise environmental control standardizes experiments resulting in more informative cross-comparison in the literature. Thus, the present approach is anticipated to have broader applicability in the research of zooplankton and fish larvae including under varied abiotic/biotic environmental conditions and aquatic contaminants.

## Supplementary Information

Supplementary Information.

## Data Availability

Datasets are available from the corresponding author on reasonable request.
