# Supplementary material for: The impact of selected abiotic factors on Artemia hatching process through real-time observation of oxygen changes in a microfluidic platform
Source: Sci Rep. 2023 Apr 19;13:6370. doi: 10.1038/s41598-023-32873-1 (PMC10115827; doi:10.1038/s41598-023-32873-1)
Supplement: Supplementary file 1 — Supplementary Information. [file 41598_2023_32873_MOESM1_ESM.pdf]

Supplementary information

The impact of selected abiotic factors on *Artemia* hatching process through real-time observation of oxygen changes in a microfluidic platform

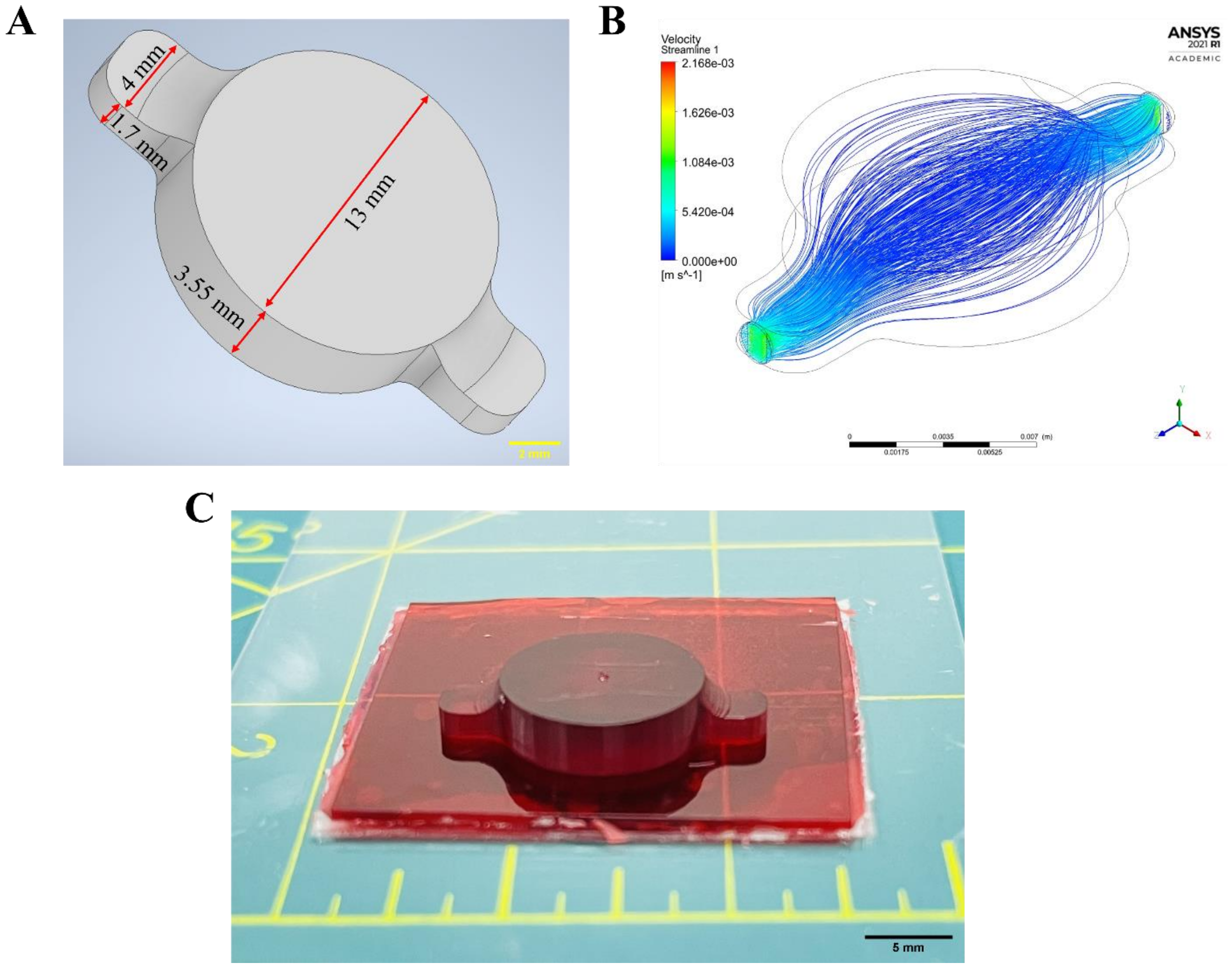

Supplementary Fig.1 (A) CAD model of the hatching chip. (B) Fluid flow simulation in the hatching chip using ANSYS FLUENT (flow rate=100 µL/min). (C) 3D printed mold for hatching chip.

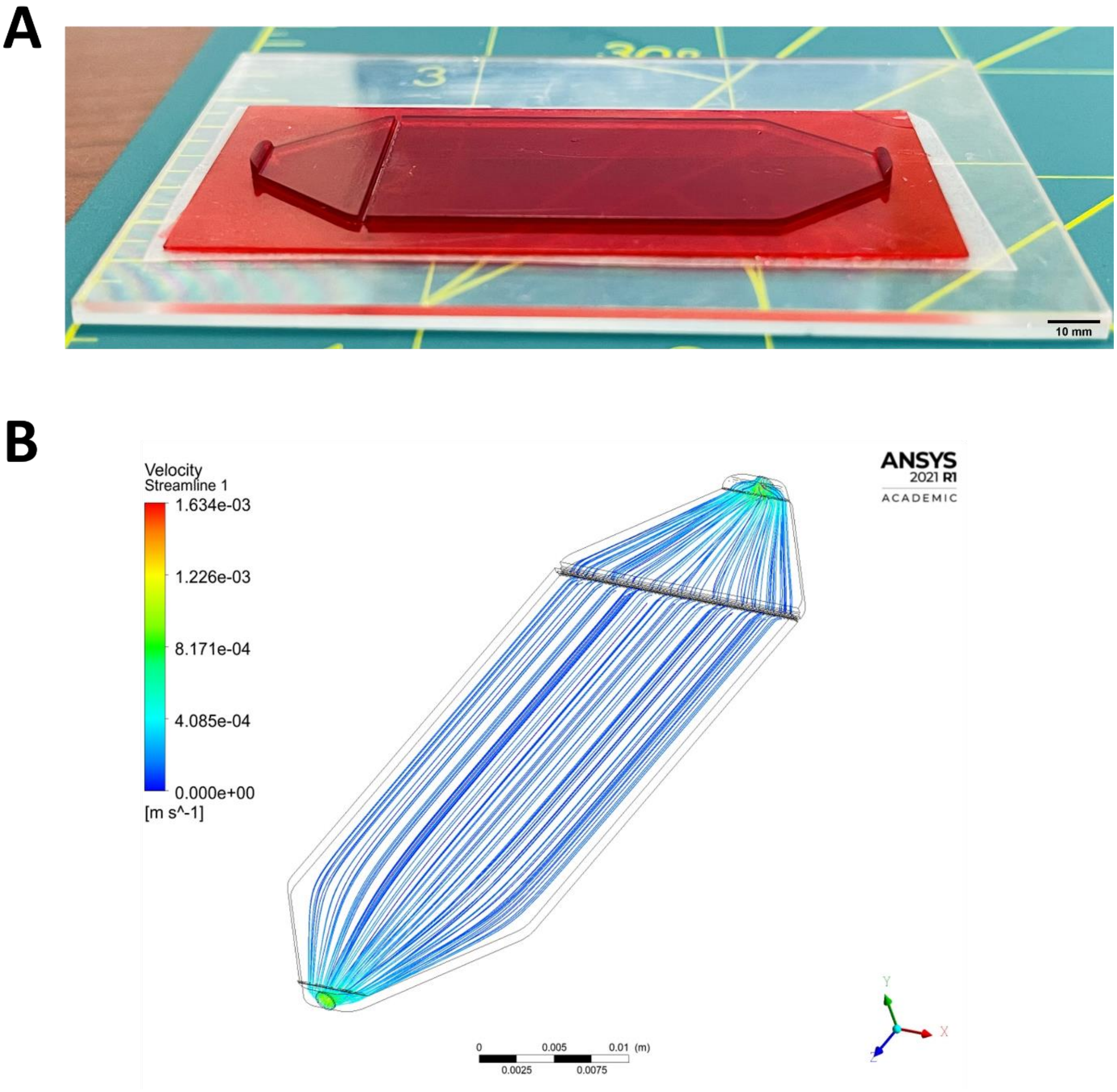

Supplementary Fig.2 (A) 3D printed mold for counting chip, (B) Fluid flow simulation of the counting chip performed on ANSYS Fluent (flow rate=100 μL/min).

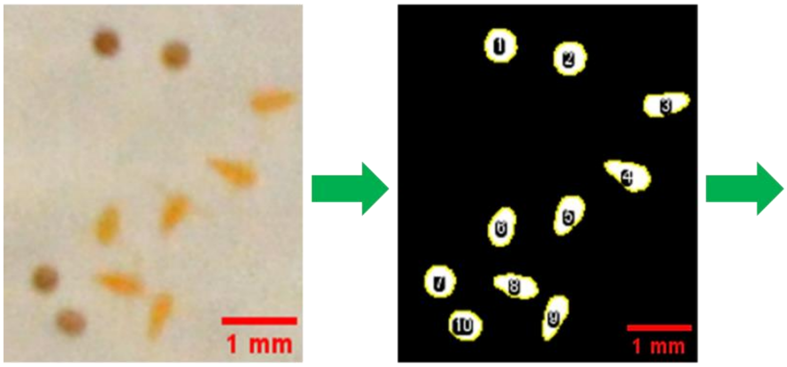

No.	Circularity (C)	C≥0.9?	Cyst/Nauplius
1	0.955	Yes	Cyst
2	0.952	Yes	Cyst
3	0.750	No	Nauplius
4	0.743	No	Nauplius
5	0.812	No	Nauplius
6	0.852	No	Nauplius
7	0.954	Yes	Cyst
8	0.745	No	Nauplius
9	0.694	No	Nauplius
10	0.986	Yes	Cyst

Number of cysts= 4
Number of nauplii= 6

Supplementary Fig.3 Estimation of the number of cysts and nauplii after hatching based on their circularity by using ImageJ program. The image depicts a subset of all cysts and nauplii, however the hatching rate was obtained by counting all the cysts and nauplii.

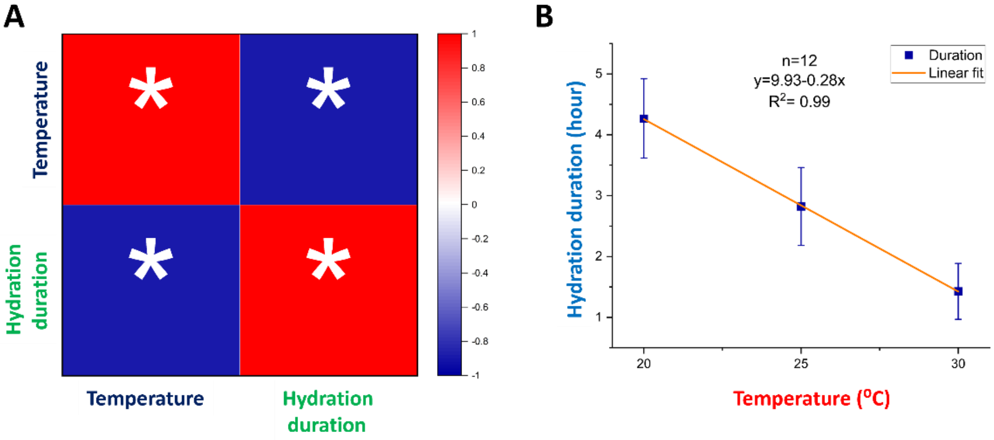

Supplementary Fig.4 Relation between temperature and hydration duration- A) Pearson correlation coefficient [n=36, * represents relation is statistically significant (p<0.05)]. B) Linear fit between hydration duration and temperature irrespective of salinity. Values are expressed as mean ± standard deviation (n=12 at each temperature point).

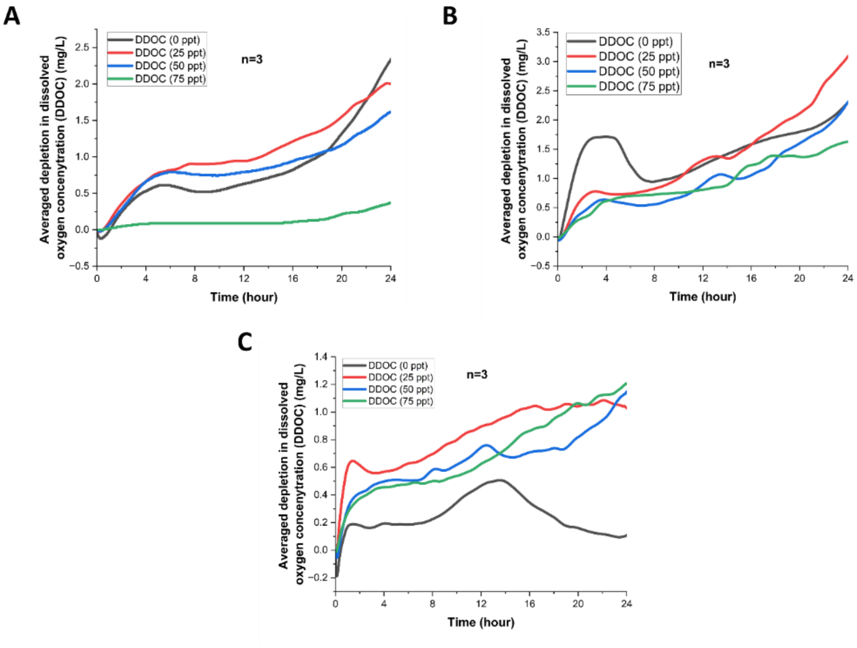

Supplementary Fig.5 Averaged changes in the depletion in dissolved oxygen concentration (DDOC) due to the oxygen consumption during hatching of *Artemia* under different salinities (0, 25, 50, and 75 ppt) and temperatures of A) 20^{0}C, B) 25^{0}C, and C) 30^{0}C (n=3).

Supplementary Table.1 ANOVA tables (* represents the data is statistically significant at p=0.05 level)

A. ANOVA table: duration of different stages

	**Hydration** (Two-way ANOVA)					**Differentiation** (Two-way ANOVA)				
	DF	Sum of Squares	Mean Square	F Value	P Value	DF	Sum of Squares	Mean Square	F Value	P Value
Salinity	3	1.04	0.35	1.15	0.35	3	250.75	83.58	61.94	1.91E-11 *
Temperature	2	48.37	24.19	79.75	2.51E-11 *	2	233.6	116.8	86.55	1.06E-11 *
Interaction	6	3.17	0.53	1.74	0.15	6	132.13	22.02	16.32	2.06E-7 *
Model	11	52.59	4.78	15.76	2.09E-08	11	616.48	56.04	41.53	6.61E-13
Error	24	7.28	0.3			24	32.39	1.35		
Corrected Total	35	59.86				35	648.86			
	**Emergence** (One-way ANOVA)					**Hatching** (One-way ANOVA)				
	Salinity					Salinity				
	DF	Sum of Squares	Mean Square	F Value	P Value	DF	Sum of Squares	Mean Square	F Value	P Value

Model	3	51.55	17.18	1.7	0.2	3	237.92	79.31	4.86	0.01 *
Error	20	202.33	10.12			20	326.62	16.33		
Total	23	253.88				23	564.54			
	Temperature					Temperature				
	DF	Sum of Squares	Mean Square	F Value	P Value	DF	Sum of Squares	Mean Square	F Value	P Value
Model	2	146.5	73.25	14.32	1.19E-4 *	2	223.84	111.92	6.9	0 *
Error	21	107.38	5.11			21	340.7	16.22		
Total	23	253.88				23	564.54			

B. ANOVA table: total oxygen consumption (Two-way ANOVA)

	**DF**	**Sum of Squares**	**Mean Square**	**F Value**	**P Value**
Salinity	3	4.36	1.45	3.42	0.03 *
Temperature	2	12.79	6.39	15.07	5.76E-5 *
Interaction	6	7.88	1.31	3.1	0.02 *
Model	11	25.02	2.27	5.36	2.91E-4
Error	24	10.18	0.42		
Corrected Total	35	35.21			

C. ANOVA table: rate of oxygen consumption (ROC) at different stages

	**Hydration** (Two-way ANOVA)					**Differentiation** (Two-way ANOVA)				
	DF	Sum of Squares	Mean Square	F Value	P Value	DF	Sum of Squares	Mean Square	F Value	P Value
Salinity	3	0.2	0.07	4.78	0.01 *	3	2.76E-4	9.18E-5	0.21	0.89
Temperature	2	0.31	0.15	10.84	4.42E-4 *	2	6.06E-4	3.03E-4	0.68	0.52
Interaction	6	0.44	0.07	5.23	0 *	6	0	7.08E-4	1.59	0.19
Model	11	0.95	0.09	6.13	1.06E-4	11	0.01	4.66E-4	1.05	0.44
Error	24	0.34	0.01			24	0.01	4.45E-4		
Corrected Total	35	1.29				35	0.02			
	**Emergence** (One-way ANOVA)					**Hatching** (One-way ANOVA)				
	Salinity					Salinity				
	DF	Sum of Squares	Mean Square	F Value	P Value	DF	Sum of Squares	Mean Square	F Value	P Value
Model	3	0.01	0	0.37	0.78	3	0.03	0.01	2.72	0.07
Error	20	0.09	0			20	0.07	0		
Total	23	0.1				23	0.1			

	Temperature					Temperature				
	DF	Sum of Squares	Mean Square	F Value	P Value	DF	Sum of Squares	Mean Square	F Value	P Value
Model	2	0.03	0.01	3.52	0.05 *	2	0.05	0.02	10.44	7.13E -4 *
Error	21	0.07	0			21	0.05	0		
Total	23	0.1				23	0.1			

D. ANOVA table: hatching rate (Two-way ANOVA)

	**DF**	**Sum of Squares**	**Mean Square**	**F Value**	**P Value**
Salinity	3	14940.71	4980.24	47.24	3.19E-10 *
Temperature	2	5642.8	2821.4	26.76	7.75E-7 *
Interaction	6	4977.08	829.51	7.87	9.28E-5 *
Model	11	25560.59	2323.69	22.04	6.59E-10
Error	24	2530.03	105.42		
Corrected Total	35	28090.62			
